# The potato tuber content of microelements as affected by organic fertilisation and production system

**DOI:** 10.1007/s10661-018-6894-x

**Published:** 2018-08-16

**Authors:** Barbara Gąsiorowska, Anna Płaza, Emilia Rzążewska, Anna Cybulska, Rafał Górski

**Affiliations:** Department of Agrotechnology, Faculty of Natural Sciences, Siedlce University of Natural Sciences and Humanities, Prusa 14, 08-110 Siedlce, Poland

**Keywords:** Potato, Microelements, Organic fertilisation, Mulch, Production system

## Abstract

The objective of the work was to determine the effect of undersown catch crops, which were either autumn-incorporated or left on the soil surface as mulch for spring incorporation, on the microelement content in the tubers of potato cultivated in the integrated and organic production system. Field research was conducted at the Zawady Experimental Farm owned by Siedlce University of Natural Sciences and Humanities, Poland (52° 03′ 39″ N, 22° 33′ 80″ E). The following two factors were examined in the study: (i) organic fertilisation, control, farmyard manure, serradella, westerwolds ryegrass, serradella applied as mulch and westerwolds ryegrass applied as mulch and (ii) production system, organic and integrated. Microelements (B, Cu, Fe, Mn, Zn) were determined in potato tubers samples. The tubers of potato manured with autumn-incorporated serradella had the highest iron and zinc contents, whereas tubers following autumn-incorporated serradella and spring-incorporated serradella mulch had the highest boron content. Manuring with undersown catch crops and farmyard manure contributed to a decline in the potato tuber content of copper and manganese. Potato cultivated in the integrated production system contained more copper, manganese and zinc, whereas organic tubers had more iron and boron.

## Introduction

Potato destined for direct consumption should have the best quality attributes (Roma et al. 2017; Sierra et al. 2017; Hajšlovǎ et al. 2005). Tuber quality is shaped by tuber chemical composition which is affected by genetic factors as well as the environment. These factors influence plant metabolism, which determines potato tuber chemical composition (Baranowska et al. 2017; Garrido et al. 2017). Organic manuring is an agrotechnological factor which beneficially affects the chemical composition of potato tubers including their content of macroelements and microelements (Wszelaki et al. 2005; Redulla et al. 2005; Griffiths et al. 2012). Farmyard manure is a major natural fertiliser applied in potato cultivation. As farmyard manure production is on the decline, and both the integrated production and organic production of potato are developing, farmers look for substitutive biomass sources. Undersown catch crops are of extreme importance in the aforementioned potato production systems (Dabney et al. 2001; Redulla et al. 2005; Snapp et al. 2005). Among the undersown catch of crops, *Fabaceae* plants are extremely valuable, which improve the biological, chemical and physical properties of the soil, including increasing the organic carbon content and the ability to exchange cations. In addition, they limit the collection of heavy metals from the soil by the potato plant; they also limit their concentration in the soil (Dabney et al. 2001; Snapp et al. 2005) In Poland, there are no studies on the impact of undersown catch crops of *Fabaceae* plants, including serradella on the content of micronutrients in potato tubers. Serradella is recommended for growing on light soils, where the cultivation of edible potato is included.

As there is a lack of Polish studies on the effect of undersown serradella on the potato tuber content of microelements, the need arises to conduct this type of research. The objective of the study reported here was to determine the impact of undersown catch crops, which were either autumn-incorporated or left on the soil surface as mulch for spring incorporation, on the concentration of microelements in the tubers of potato cultivated in the integrated and organic production systems.

## Materials and methods

Field research spanned the years 2008–2011 and was conducted at the Zawady Experimental farm which belongs to Siedlce University of Natural Sciences and Humanities. The experimental soil was albic luvisol representing the IVa soil valuation class. Available minerals in the soil were as follows: P 5.18 mg 100 g^−1^ (spectrophotometric method), K 11.29 mg 100 g^−1^ (flame photometry method), Mg 5.43 mg 100 g^−1^(flame atomic absorbtion spectroscopy (FAAS)), Mn 107 mg 100 g^−1^ (FAAS), Cu 1.2 mg 100 g^−1^ (FAAS), B 0.50 mg 100 g^−1^ (spectrophotometric method), Zn 7.1 mg 100 g^−1^ (FAAS) and Fe 649 mg 100 g^−1^ (FAAS). Soil reaction was neutral, and humus content was 1.37%. An experiment was a split-block arrangement of plots with three replicates. The following two factors were examined in the study: (i) organic fertilisation, control (no manuring with undersown catch crop), farmyard manure (30 tּha^−1^), serradella (22.3 tּha^−1^), westerwolds ryegrass (35.4 tּha^−1^), serradella applied as mulch (22.5 tּha^−1^) and westerwolds ryegrass applied as mulch (35.5 tּha^−1^) and (ii) production system, organic and integrated.

Spring triticale grown for grain was undersown with catch crops and followed by table potato. In the integrated production system, mineral fertilisers were applied to the whole experimental area in early spring. Their amounts were as follows: 90 kg N, 36.9 kg P and 99.6 kg K. Nitrogen was used in the form of ammonium nitrate, phosphorus in the form of superphosphate and potassium in the form of potassium sulphate. Fertiliser rates were adjusted to soil availability and amounts of projected yields. In plots which had been ploughed in autumn, in spring fertilisers were mixed with soil using a cultivator with a harrow attached to it. In mulched units, a disc harrow was followed by a cultivator. In the organic production system, mineral fertilisation was replaced with farmyard manure applied to the whole experimental area at the rate of 30 tּha^−1^ and followed by spring triticale undersown with catch crops. Potatoes were planted in late April and harvested in mid-September. The Zeus potato varieties were cultivated. This is a mid-late variety. In Poland, it is recommended for organic farming because it is quite resistant to *Phytophthora infestans*. In the integrated production system, mechanical and chemical control of weeds, pests and diseases was used. Potatoes were hilled and then harrowed every 7 days up to emergence. Just before potatoes emerged, they were sprayed with a mixture of the herbicides Afalon 50 WP (linuron 450 gּ dm^3^) + Reglone 200 SL (dibromide diquat 3 dm^3^ ּha^−1^). Colorado potato beetle was controlled using two applications of Fastac 100 EC (alpha–cypermethrin 0.1 dm^3^ ּha^−1^) and late potato blight using two applications of the fungicide Ridomil GOLD MZ PEPITE 67,8 WG (metalaxyl-M, mancozeb 2 kgּ ha^−1^). Weeds in the organic crop were removed mechanically. Colorado potato beetle was controlled by means of two applications of Novodor SC (*Bacillus thuringiensis* subspecies *tenebrionis* 2.5 dm^3^ ּha^−1^) and late potato blight using three applications of the fungicide Miedzian 50 WP (copper oxychloride 4 kgּ ha^−1^). During potato harvest, tuber samples were collected from each plot to determine microelements. Samples of tubers of various sizes (approximately 20) were collected from each plot in accordance with the proportional share in the yield. Tubers were crumbled in a blender. The crushed pulp was placed on a petriego dish and dried in an air dryer, at a temperature not exceeding 60 °C, to an air-dry condition. The dried material was grind in a planetary-ball mill Retsch PM 100, resulting in final fineness below 1 mm. Cu, Fe, Mn, Zn and B contents were determined in the dry matter of tubers by means of inductively coupled plasma atomic emission spectroscopy (ICP-OES). Then the plant material was taken up in acids in a microwave digestor Milesone Ethos Plus. The content of micronutrients was determined in the mineralisation by emission spectrometry with excitation in inductively coupled plasma and an optical detector (ICP-OES), using an emission spectrometer Perkin Elmer Optima 8300. The assay was performed in triplicate.

Each of the characteristics studied was analysed by means of ANOVA for the split-block arrangement. Comparison of means for significant sources of variation was achieved by means of Tukey’s test at the significance level of *P* ≤ 0.05. All the calculations were performed in STATISTICA®, version 12.0, and MS Excel.

The years of conducting the research were characterised by a varied course of weather conditions, especially the sum and distribution of precipitation in the growing seasons of potato (Table 1). In 2010, the highest sum of precipitation was highest at the highest average temperature, which adversely affected the content of micronutrients in potato tubers. The year 2009 turned out to be more beneficial for collecting micronutrients in potato tubers, because a smaller sum of rainfall was recorded, but a lower average temperature. By contrast, in 2011, the smallest sum of precipitation was recorded, at medium temperature approximated to the long-term average, which had a positive effect on the accumulation of micronutrients in potato tubers.

**Table 1:** Weather conditions in the growing season of potato according to the Zawady Metorological Station

Year	Month						Mean
	April	May	June	July	August	September	
Mean air temperature, °C							
2009	10.3	12.9	15.7	19.4	17.7	14.6	15.1
2010	8.9	14.0	17.4	21.6	19.8	11.8	15.6
2011	10.1	13.4	18.1	18.3	18.0	14.4	15.4
Long-term (15 years) mean	8.2	14.2	17.6	19.7	19.1	12.9	15.3
Rainfall sum, mm							
2009	8.1	68.9	145.2	26.4	80.9	24.9	354.4
2010	10.7	93.2	62.6	77.0	106.3	109.9	459.7
2011	31.0	36.1	39.1	120.2	18.6	12.0	257.0
Long-term (15 years) mean	37.4	47.1	48.1	65.5	43.5	47.3	288.9

## Results and discussion

Statistical analysis demonstrated a significant effect of the experimental factors and their interaction on boron content in potato tubers (Table 6). The lowest boron content (4470 mg ּkg^−1^) was determined in the tubers of control potatoes following mineral fertiliser only. This finding agrees with results reported by Sayed et al. (2015). In the experiment presented here, green manures and farmyard manure increased the potato tuber content of boron. The highest concentration (5514–5404 mg ּkg^−1^) of this element was determined in the tubers of potato manured with both autumn-incorporated serradella and spring-incorporated serradella mulch. Boron content in the tubers of potato following westerwolds ryegrass, regardless of when it had been incorporated, was significantly lower (4994–4880 mg ּkg^−1^) than in tubers harvested in farmyard manure-amended units (5185 mg kg^−1^), it being higher compared with control potato (4470 mg kg^−1^). Also research by Snapp et al. (2005) demonstrated that green manures and vermicompost contributed to an increase in the concentration of boron in potato tubers. In the work reported here, production system had a significant impact on the potato tuber content of boron. This content was higher in the potato tubers produced using organic (5150 mg ּkg^−1^) rather than integrated farming practices (4998 mg ּkg^−1^). Similar results were also obtained by Hajšlovǎ et al. (2005) and Wierzbowska et al. (2018). In the study discussed here, there was observed an interaction of the experimental factors which indicated that the highest boron content (5617 mg ּkg^−1^) was characteristic of tubers of organic potato following autumn-incorporated serradella, and the lowest (4415 mg ּkg^−1^) in the tubers of control potato following mineral fertiliser only in the integrated production system.

Statistical analysis demonstrated a significant effect of manuring with undersown catch crops, production system and their interaction on the potato tuber content of copper (Table 2). The highest copper content was determined in control tubers grown on farmyard manure only (4683 mgּ kg^−1^). This finding corresponds to results reported by Braun et al. (2011) and Roma et al. (2017) who obtained a higher copper content in the tubers of potato fertilised with mineral fertiliser. In the present study, an application of farmyard manure and undersown catch crops prior to potato cultivation contributed to a significant decline in the potato tuber content of copper. The lowest copper concentration was determined in the tubers of potato following autumn-incorporated undersown serradella (4405 mg kg^−1^). In the remaining plots manured with undersown catch crops, the potato tuber content of copper differed insignificantly from the concentration of this micronutrient in farmyard manure-amended units. In the research by Musilova et al. (2016) and Garrido et al. (2017), the authors have demonstrated that manuring of potato with vermicompost and green manures contributes to a decline in the potato tuber content of copper. In the study reported here, production system affected the copper content in potato tubers. The concentration of this element was the lowest in organic tubers (4390 mg ּkg^−1^) compared with the integrated system (4609 mg ּkg^−1^). A similar finding has been reported by Hajšlovǎ et al. (2005), Wang et al. (2008) and Hunter et al. (2011). Whereas in the studies by Wierzbowska et al. (2018), the ecological production system has a higher content of Cu (2.8–3.1 mg ּkg^−1^) than in an integrated and conventional production system. In the experiment reported here, there was observed an interaction between the factors studied. The lowest copper content (4313–4394 mg ּkg^−1^) was recorded in the tubers of organic potato manured with undersown catch crops and farmyard manure, it being the highest in the tubers harvested in the control unit in the integrated production system (4844 mg ּkg^−1^) where only mineral fertilisation had been applied.

**Table 2:** Copper content in potato tubers (means over 2009–2011), mgּ kg^−1^ dm

Organic fertilisation	Production system		Means
	Integrated	Organic	
Control	4.844	4.522	4.683
Farmyard manure	4.609	4.394	4.502
Serradella	4.496	4.313	4.405
Westerwolds ryegrass	4.598	4.372	4.485
Serradella—mulch	4.502	4.360	4.431
Westerwolds ryegrass—mulch	4.607	4.381	4.494
Means	4.609	4.390	–
LSD_0.05_			
Organic fertilisation			0.073
Production system			0.068
Interaction			0.090

The iron content in potato tubers was significantly affected by the experimental factors and their interaction (Table 3). The lowest concentration of iron was determined in the tubers harvested in the control unit where only mineral fertilisation had been applied. Similarly, Braun et al. (2011), Hadi et al. (2014) and Ashrafzadeh et al. (2017) reported the lowest iron content in the tubers of potato fertilised with mineral fertiliser only. In the present study, farmyard manure and undersown catch crops contributed to an increase in the potato tuber content of iron, which may have occurred due to the fact that farmyard manure and green manures introduce organic substance an additional amount of macroelements and microelements into the soil which results in an increase of Fe collection by potato plants of valuable microelement for human health (Braun et al. 2011; White et al. 2009; Sierra et al. 2017). In the work reported here, the highest iron content was recorded in the tubers of potato manured with autumn-incorporated serradella. Iron content (53.14 mg ּkg^−1^) in the tubers of potato manured with serradella mulch differed insignificantly from iron concentration in the tubers of potato harvested from farmyard manure-amended units. Only after westerwolds ryegrass, regardless of when it had been applied, the iron content was significantly lower compared with tubers harvested from farmyard manure-amended plots, it still being higher than in control tubers. Production system significantly influenced the potato tuber content of iron which was higher in the tubers of organic potato (48.10 mg ּkg^−1^) compared with the integrated production system (46.57 mg ּkg^−1^). This finding corresponds with reports by Wierzbowska et al. (2018). In the study discussed here, there was confirmed an interaction of the experimental factors which revealed that the highest (53.71–52.56 mg ּkg^−1^) iron concentration was determined in the tubers of potato following autumn-incorporated serradella in the organic and integrated production systems, it being the lowest (41.09 mg ּkg^−1^) in the tubers of control potato produced using integrated farming practices which included mineral fertiliser application.

**Table 3:** Iron content in potato tubers (means over 2009–2011), mg ּkg^−1^ dm

Organic fertilisation	Production system		Means
	Integrated	Organic	
Control	41.09	43.14	42.12
Farmyard manure	49.50	50.94	50.22
Serradella	52.56	53.71	53.14
Westerwolds ryegrass	44.14	45.22	44.68
Serradella—mulch	49.03	51.10	50.07
Westerwolds ryegrass—mulch	43.09	44.51	43.80
Means	46.57	48.10	–
LSD_0.05_			
Organic fertilisation			1.24
Production system			0.80
Interaction			1.38

Statistical analysis demonstrated a significant effect of the experimental factors and their interaction on the potato tuber content of manganese (Table 4). The highest concentration (8505 mg ּkg^−1^) of this element was recorded in the tubers of control potato following mineral fertiliser. Also White et al. (2009), Braun et al. (2011), Hadi et al. (2014), Ashrafzadeh et al. (2017) and Baranowska et al. (2017) obtained the highest manganese content in the tubers of potato following mineral fertiliser. In the present study, green manures and farmyard manure contributed to a significant decline in the potato tuber of manganese, which agrees with findings reported by Redulla et al. (2005) as well as Roma et al. (2017). Also Baranowska et al. (2017) and Öborn et al. (1995) demonstrated that green manures that enrich the soil with an organic substance application reduced manganese content in potato tubers. In the study reported here, manganese content in the tubers of potato following undersown catch crops, whether they had been applied in autumn or spring, was significantly lower (7951–8212 mg ּkg^−1^) compared with potato following farmyard manure (8313 mg ּkg^−1^). Production system had a significant effect on the potato tuber content of manganese. Similarly to the works by Wszelaki et al. (2005) and Sawicka et al. (2016), the concentration of this element was higher in the tubers of potato produced using integrated farming (8261 mg ּkg^−1^) practices compared with organic production system (8118 mg ּkg^−1^). There was confirmed an interaction between the experimental factors which indicated that the lowest manganese content was determined in the tubers of organic potato following serradella (7888–7957 mg kg^−1^), regardless of the time of the undersown catch crop incorporation, it being the highest (8621 mg kg^−1^) in control tubers produced using integrated farming practices. Also in the studies of Wierzbowska et al. (2018), the content of Mn in potato tubers cultivated in an organic production system was lower (6–6.4 mg kg^−1^) than in an integrated and conventional production system.

**Table 4:** Manganese content in potato tuber (means over 2009–2011), mg ּkg^−1^ dm

Organic fertilisation	Production system		Means
	Integrated	Organic	
Control	8.621	8.388	8.505
Farmyard manure	8.406	8.219	8.313
Serradella	8.013	7.888	7.951
Westerwolds ryegrass	8.201	8.094	8.148
Serradella—mulch	8.072	7.957	8.015
Westerwolds ryegrass–mulch	8.261	8.163	8.212
Means	8.262	8.118	–
LSD_0.05_			
Organic fertilisation			0.079
Production system			0.075
Interaction			0.091

Zinc content in potato tubers was significantly affected by the experimental factors and their interaction (Table 5). The lowest zinc (9.86 mg ּkg^−1^) concentration was recorded in the tubers of control potato produced in mineral fertiliser-amended plots. In their study, Braun et al. (2011), Hadi et al. (2014) and Ashrafzadeh et al. (2017) who found the lowest zinc content in the tubers of potato following mineral fertiliser. In the present study, there was observed an increase in the zinc content in the tubers of potatoes following farmyard manure and undersown catch crops which are an abundant source of organic substances, macroelements and microelements (10.98–12.47 mg ּkg^−1^). This finding corresponds to the results reported by Öborn et al. (1995) and Mengist et al. (2018) who have demonstrated that green manure application contributes to an increase in the potato tuber content of zinc. In the study discussed here, the highest zinc content (12.47 mg ּkg^−1^) was determined in the tubers of potato following autumn-incorporated serradella. In the remaining undersown catch crop-amended units, excluding westerwolds ryegrass left on the soil surface as mulch for spring incorporation, the potato tuber content of zinc differed insignificantly from the content determined in the tubers of potato following farmyard manure. Production system significantly affected the potato tuber content of zinc. Its concentration was higher in the tubers of potato produced using integrated farming practices (11.81 mg ּkg^−1^). Such a relationship was also observed in the studies by Hajšlovǎ et al. (2005) and Wang et al. (2008). In the present study, there was confirmed an interaction of the experimental studied which indicated that the highest zinc content (13.23–12.48 mg ּkg^−1^) was recorded in the tubers of potato manured with autumn- and spring-incorporated serradella in the integrated production system, and the lowest (9.44 mg ּkg^−1^) in the tubers of control organic potato (Table 6).

**Table 5:** Zinc content in potato tuber (means over 2009–2011), mg ּkg^−1^ dm

Organic fertilisation	Production system		Means
	Integrated	Organic	
Control	10.28	9.44	9.86
Farmyard manure	11.90	11.03	11.47
Serradella	13.23	11.70	12.47
Westerwolds ryegrass	11.60	11.23	11.42
Serradella—mulch	12.48	11.77	12.13
Westerwolds ryegrass—mulch	11.37	10.59	10.98
Means	11.81	10.96	–
LSD_0.05_			
Organic fertilisation			0.65
Production system			0.46
Interaction			0.85

**Table 6:** Boron content in potato tuber (means over 2009–2011), mg ּkg^−1^ dm

Organic fertilisation	Production system		Means
	Integrated	Organic	
Control	4.415	4.524	4.470
Farmyard manure	5.117	5.253	5.185
Serradella	5.411	5.617	5.514
Westerwolds ryegrass	4.911	5.077	4.994
Serradella—mulch	5.326	5.481	5.404
Westerwolds ryegrass—mulch	4.809	4.950	4.880
Means	4.998	5.150	–
LSD_0.05_			
Organic fertilisation			0.059
Production system			0.041
Interaction			0.082

It is necessary to assess food safety in relation to contaminants present in edible parts, including potato tubers. Monitoring the content of toxic and potentially toxic elements is one of the most important aspects of food quality assurance. The conducted research has shown that the use of green manure and manure increases the content of organic matter in the soil, which limits the uptake of elements harmful to human health. In particular, the serradella is valuable here, both ploughed in autumn and left to spring in the form of a mulch used in potato fertilisation.

## Conclusions

The tubers of potato manured with autumn-incorporated serradella had the highest iron and zinc contents, and tubers of potato following serradella, either autumn-incorporated or left on the soil as mulch for spring incorporation, had the highest boron content. Manuring with undersown catch crops and farmyard manure contributed to a decline in copper and manganese contents in potato tubers.

Potato produced using integrated farming practices contained more copper, manganese and zinc, whereas organic crop had more iron and boron.

The highest concentrations of microelements, excluding copper and manganese, were determined in the tubers of potato following serradella, regardless of when it had been applied, cultivated in the integrated and organic production systems.
